# Wernicke-Korsakoff Syndrome as a Consequence of Hyperemesis Gravidarum: A Case Report

**DOI:** 10.7759/cureus.42766

**Published:** 2023-07-31

**Authors:** Haniel B Souza, Rafaela F Gonçalves, Monteiro J Moreira, Rodrigo L Tavares, Gustavo R Isolan

**Affiliations:** 1 Department of Neurology, Universidade Federal do Rio Grande do Sul (UFRGS), Porto Alegre, BRA; 2 Department of Neurology, The Center For Advanced Neurology and Neurosurgery, Brazil (CEANNE), Porto Alegre, BRA; 3 Department of Neurosurgery, The Center for Advanced Neurology and Neurosurgery, Brazil (CEANNE), Porto Alegre, BRA

**Keywords:** vitamin b1 deficiency, nausea and vomiting in pregnancy, neurology, clinical case report, thiamine deficiency, hyperemesis gravidarum, wernicke-korsakoff syndrome

## Abstract

Wernicke-Korsakoff syndrome (WKS) is caused by severe thiamine (vitamin B1) deficiency and can lead to chronic deficits. In this case, a 22-year-old pregnant patient at 10 1/7 weeks of gestation presented to the emergency department with malaise, asthenia, headache, weakness, vomiting, and weight loss of 12 kg. Pancreatitis and hepatic steatosis were considered but ruled out, and cholecystolithiasis was confirmed by ultrasound. After significant neurological deterioration, the patient underwent a cranial MRI that revealed suggestive findings in the thalamus consistent with WKS. WKS is a rare complication of hyperemesis gravidarum and should be included in the differential diagnosis of persistent vomiting in order to initiate early and appropriate treatment.

## Introduction

During pregnancy, women undergo profound physiological and emotional changes that can impact maternal and child health. Some factors associated with a better quality of life, such as favorable social and economic conditions, family support, and engaging in physical activities, can be considered protective factors. On the other hand, factors such as obesity, body pain, stress, anxiety, sleep difficulties, history of alcohol consumption, smoking, and poor diet can be considered aggravating factors for maternal and infant health [1].

During gestation, a mother´s body demands more nutrients for fetal development. Therefore, a diet rich in vitamins and minerals is important. Thiamine, also known as vitamin B1, is a fundamental micronutrient as it acts as a critical coenzyme for the synthesis of essential neurotransmitters for brain function, as well as a neuromodulator of acetylcholine [2].

Thiamine deficiency can cause neuronal damage and inhibition of metabolism in certain areas of the brain like the thalamus, mammillary bodies, tectal plate, and periaqueductal areas [3], generating cytotoxic edema, blood-brain barrier breakdown [4] and leading to serious manifestations such as Wernicke-Korsakoff syndrome (WKS) [5].

WKS is mainly caused by malabsorption of thiamine or its deficiency, which occurs in alcoholics, malnourished individuals, and those with anorexia nervosa, bariatric surgery, and neoplasms. Women in the first trimester of pregnancy suffering from thiamine deficiency are at risk of developing hyperemesis gravidarum. This syndrome is not as rare as previously thought, though among non-alcoholics the prevalence varies from 0.04% to 0.13% [6,7].

This syndrome manifests in two distinct phases: initially, but not always, Wernicke's encephalopathy (WE) (the acute phase of the syndrome) arises as is characterized by the classic clinical triad of acute confusional state, ophthalmoplegia or nystagmus, and ataxia. With the progression of the pathological process, encephalopathy can progress to Korsakoff's syndrome (a chronic condition) marked by anterograde amnesia and confabulation. Generally, WE is reversible, but the late identification and treatment of this syndrome can lead to coma and death. Complications for pregnant women and fetuses are more severe, causing permanent neurological deficits in mothers and even death in 10-20% of cases. It can also cause spontaneous abortion, premature delivery, and intrauterine growth restriction [8,6].

In this study, we report the case of a young woman who was admitted to a hospital in Brazil.

## Case presentation

**Figure 1:**
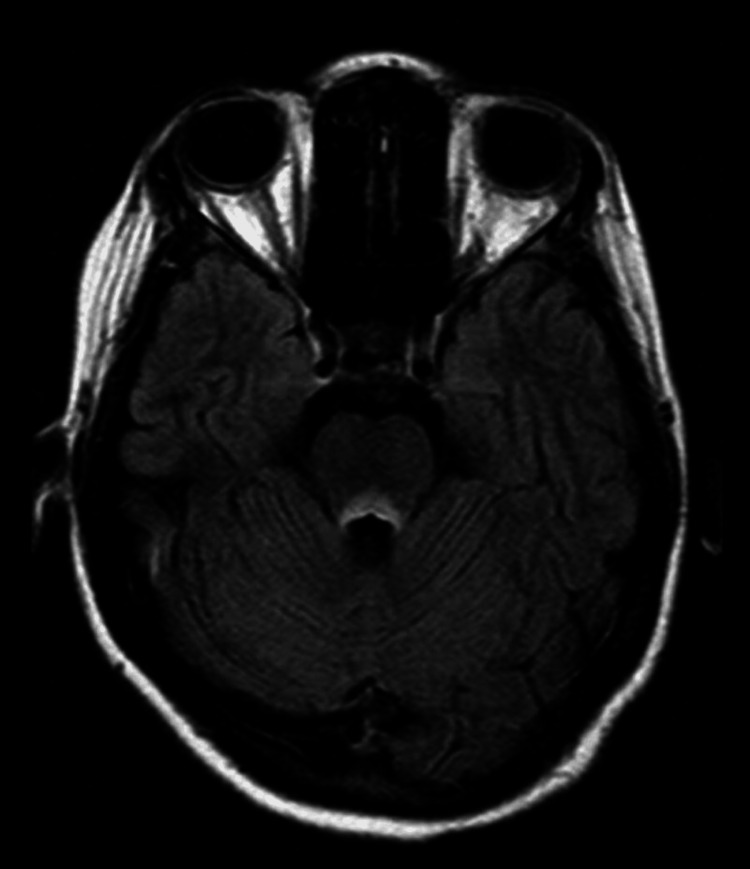
Axial T2-FLAIR MRI showed areas of symmetrical increased signal involving the mammillary bodies, dorsomedial thalami, tectal plate, periaqueductal area and/or around the third ventricle. FLAIR: fluid-attenuated inversion recovery

**Figure 2:**
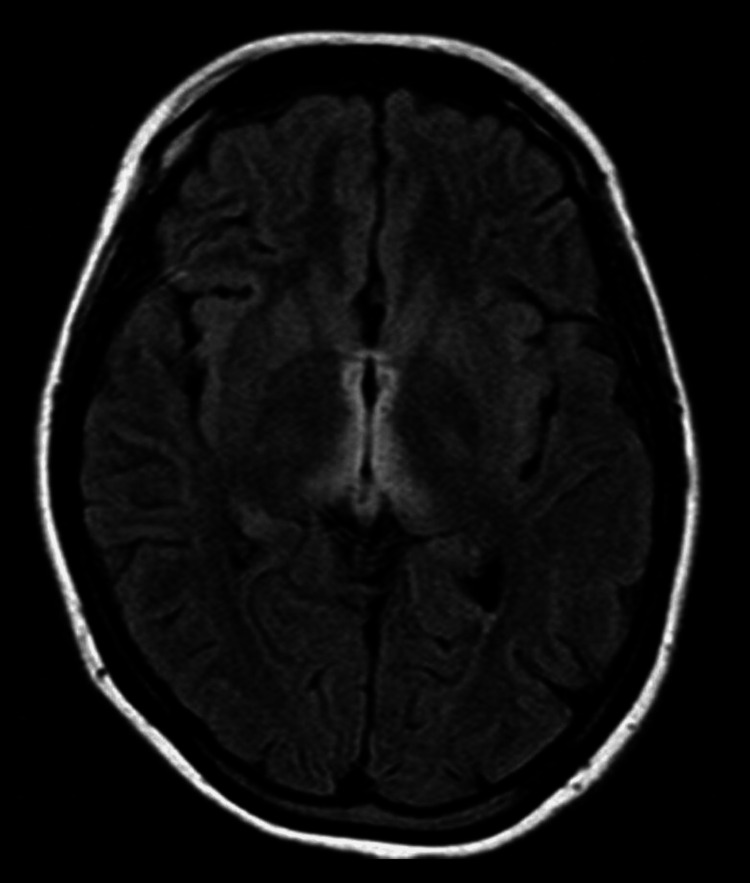
T1-weighted and DWI images showed no alterations DWI: diffusion weighted imaging

**Figure 3:**
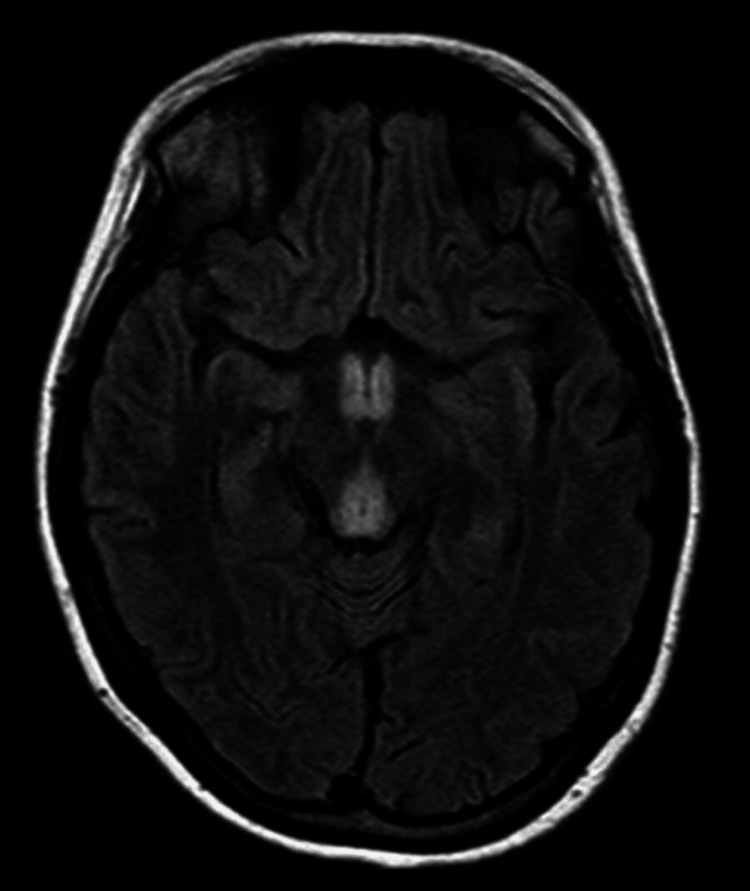
FLAIR MRI showed areas of symmetrical increased signal involving the mammillary bodies, dorsomedial thalami, tectal plate, periaqueductal area and/or around the third ventricle T1 weighted (with or without gadolinium) and DWI images showed no alterations. FLAIR: fluid-attenuated inversion recovery; DWI: diffusion weighted imaging

A 22-year-old woman at 10 weeks and one day (10+1) of gestation, previously healthy, sought medical attention on March 25, 2022, due to asthenia, headache, weakness, vomiting associated with a weight loss of 12 kg, and poor appetite. The patient was admitted for investigation and upon initial exams was found to be in good general condition, lucid, hypochromic, afebrile, and without other changes. Laboratory tests such as serum electrolytes, urine ketones, creatinine, complete blood count, amylase/lipase, and thyroid function tests were requested, which came back normal. Symptomatic medications were started with antiemetics such as dimenhydrinate, ondansetron, and promethazine, as well as good hydration. The patient presented a deterioration of her condition with progressive impairment of consciousness on April 7, 2022, and was noticed to have strabismus, nystagmus, speech disorder, and only responding to painful stimuli, but with stable vital signs. She underwent cranial MRI (Figures 1-3).

After reviewing the MRI results, supplementation with injectable Citoneurin 5000 (thiamine 100 mg + pyridoxine 100 mg + cyanocobalamin 5000 mcg) every eight hours, and injectable B complex IV every 12 hours diluted in 0.9% saline solution was started, along with the placement of a nasoenteral tube for enteral nutrition. The patient's condition progressed to intrauterine death and a cesarean section was performed. After the introduction of drug therapy, strabismus improved but nystagmus and speech disorder continued. The patient started motor and respiratory physiotherapy and continued to be monitored by gynecologists and neurologists through her progressive clinical improvement.

After resolution of the condition, the patient was discharged with a follow-up appointment. At the outpatient visit, she presented without any sequelae or complications from her previous condition. However, she demonstrated a constant feeling of fear and concern that something bad might happen to her (anxiety), in need of psychological support.

## Discussion

The body naturally stores 25-30 mg of water-soluble thiamine, which can last for about 18 days. Hyperemesis gravidarum can lead to constant vomiting and is considered common during pregnancy. However, WKS is rare and can occur at 14-18 weeks of gestation after two to three weeks of vomiting.

Physical manifestations, MRI findings, and rapid improvement of symptoms with thiamine replacement are what define diagnosis, with the main criteria being (i) dietary changes, (ii) ocular disorders, (iii) cerebral dysfunction, and (iv) memory impairment [7].

Hyperemesis gravidarum has different etiologies [8] that can originate from liver dysfunction or thyroid dysfunction, and biochemical tests for the function of these organs, such as thyroid stimulating hormone (TSH), T3, T4, albumin, and bilirubin, are important. WKS can be easily diagnosed after laboratory measurement of thiamine, but the test is not routine in the Brazilian healthcare system [9].

Imaging tests also become important for the diagnosis of this syndrome. MRI is currently considered the most valuable method to confirm the diagnosis of WE. Findings include bilateral and symmetrical increased T2 signals in the paraventricular regions of the thalamus, hypothalamus, mammillary bodies, periaqueductal region, floor of the fourth ventricle, and cerebellum [10].

Treatment for this syndrome is well established and therefore based on correcting electrolyte imbalance and dehydration and intravenous thiamine administration (200mg/day for two days), in combination with other B vitamins [11] which can result in symptom resolution in a few hours or days depending on the severity of the disease. However, rigorous studies are lacking to confirm the efficacy of this treatment [9,12].

It is not uncommon for patients to have continuing symptoms such as nystagmus, memory impairment, and ataxia after developing WKS. There are no medicinal recommendations or effective treatments for neurocognitive recovery in patients who have had the syndrome [9].

## Conclusions

The patient here presents a rare case of hyperemesis gravidarum that culminates in WKS, making it essential to observe warning signs. In the presence of constant and uninterrupted vomiting, a specialist physician should be sought, and a balanced diet and vitamin supplementation should be provided. The onset of symptoms and clinical manifestation should be carefully observed, as they, along with laboratory tests, lead to an accurate diagnosis and early and appropriate treatment, thus reducing the chances of adverse events.
